# Degenerate Wave and Capacitive Coupling Increase Human MSC Invasion and Proliferation While Reducing Cytotoxicity in an *In Vitro* Wound Healing Model

**DOI:** 10.1371/journal.pone.0023404

**Published:** 2011-08-16

**Authors:** Michelle Griffin, Syed Amir Iqbal, Anil Sebastian, James Colthurst, Ardeshir Bayat

**Affiliations:** 1 Plastic & Reconstructive Surgery Research, School of Translational Medicine, Manchester Interdisciplinary Biocentre, University of Manchester, Manchester, United Kingdom; 2 Fenzian Limited, Hungerford, Berkshire, United Kingdom; 3 Department of Plastic and Reconstructive Surgery, South Manchester University Hospital Foundation Trust, Wythenshawe Hospital, Manchester, United Kingdom; 4 Manchester Academic Health Science Centre, South Manchester University Hospital Foundation Trust, Wythenshawe Hospital, The University of Manchester, Manchester, United Kingdom; University of Medicine and Dentistry of New Jersey, United States of America

## Abstract

Non-unions pose complications in fracture management that can be treated using electrical stimulation (ES). Bone marrow mesenchymal stem cells (BMMSCs) are essential in fracture healing; however, the effect of different clinical ES waveforms on BMMSCs cellular activities remains unknown. We compared the effects of direct current (DC), capacitive coupling (CC), pulsed electromagnetic field (PEMF) and degenerate wave (DW) on cellular activities including cytotoxicity, proliferation, cell-kinetics and apoptosis by stimulating human-BMMSCs 3 hours a day, up to 5 days. In addition, migration and invasion were assessed using fluorescence microscopy and by quantifying gene and protein expression. We found that DW had the greatest proliferative and least apoptotic and cytotoxic effects compared to other waveforms. DC, DW and CC stimulations resulted in a higher number of cells in S phase and G_2_/M phase as shown by cell cycle analysis. CC and DW caused more cells to invade collagen and showed increased MMP-2 and MT1-MMP expression. DC increased cellular migration in a scratch-wound assay and all ES waveforms enhanced expression of migratory genes with DC having the greatest effect. All ES treated cells showed similar progenitor potential as determined by MSC differentiation assay. All above findings were shown to be statistically significant (p<0.05). We conclude that ES can influence BMMSCs activities, especially DW and CC, which show greater invasion and higher cell proliferation compared to other types of ES. Application of DW or CC to the fracture site may help in the recruitment of BMMSCs to the wound that may enhance rate of bone healing at the fracture site.

## Introduction

Non-union and delayed union bone fractures pose difficult complications for surgeons in approximately 5–10% of cases [1]. Autologous bone grafts have been the traditional mode of treatment for non-unions. However, the significant problems with the harvesting procedure and limited supply of autologous bone grafts, cell-based strategies have been developed recently to tackle this challenging clinical problem [2], [3]. Mesenchymal stem cells (MSCs) are often the first choice for treating non-unions since they are naturally present in the bone marrow as well as show high proliferative capacity and are able to give rise to osteocytes and chondrocytes [4].

The process of bone regeneration during healing is comparable to embryonic development with MSCs playing a vital role [5]. Within the first twenty-four hours of a fracture, cytokines and growth factors are released by platelets, macrophages and other inflammatory cells to recruit MSCs from the periosteum and the bone marrow to the fracture site. MSCs then proliferate and differentiate into osteoblasts and chondrocytes, which can contribute to bone and callus formation at the fracture site [5], [6]. In order to employ cells to the wound site and to enhance their invasive and proliferative capacity, electrical stimulation (ES) has been used since 1841 when ‘shocks of electric fluid’ were found to successfully treat non-unions [7]. Later Yasuda demonstrated that bone displays piezoelectric properties that encouraged the use of exogenous ES to enhance fracture healing [8]. In recent years, three ES waveforms have been used clinically to repair bone including direct current (DC), capacitive coupling (CC) and pulsed electromagnetic field (PEMF) stimulation. PEMF has been shown to enhance proliferation, promote the genes associated with differentiation and have the same potential for mineralization compared to unstimulated MSCs [9]. However, the effect of other waveforms on MSCs cellular activity remains unknown including MSCs invasive and migratory capacity.

One of the most remarkable yet least understood mechanisms of MSCs is their ability to migrate and become integrated into tissues for growth and repair [10]. Animal models have illustrated that following systemic injection, MSCs migrate to injured tissues to promote healing in myocardial infarction, ischemic brain injury, bone fractures and muscular dystrophy [11], [12], [13], [14].

Another requirement for cells to reach distant organs is the ability to traverse through the extracellular matrix (ECM) [15]. In order to determine whether ES promotes cell invasion, we carried out migration and collagen invasion assays. We also looked into proteolytic enzymes that are released at the wound site and thought to help in mobilization of migrating cells. In particular, we investigated matrix-metalloproteinases (MMP)-2 and MMP-9, the gelatinases, which degrade denatured collagens, laminin and collage type IV components of the ECM. Additionally, they have been shown to be linked with the invasive capacity of tumor cells and leukocytes [15]. MMP-2 requires activation by proteolytic removal of the N-terminal proenzyme domain, which was shown to be executed by membrane-type-1 MMP (MT1-MMP) [16]. Recent studies have illustrated the invasive capacity of human MSCs requiring MMP-2 and MT1-MMP [17], [18].

We have previously demonstrated the beneficial effect of DW on skin fibroblasts [19], and we have hypothesized that DW could also enhance MSCs proliferation and hence its effectiveness as bone healing cell. We have stimulated cells for 3 hrs/day for up to 5 days since our study also showed that fibroblasts could be propagated after ES treatment for up to 12 hrs [19]. Therefore in this study, we have compared the effects of various ES waveforms including PEMF, DC, CC and DW on the cellular activities of bone marrow mesenchymal stem cells (BMMSCs). We provide evidence that ES increases the ability of BMMSCs to proliferate, invade and migrate to varying extents depending on the exact mode of electrical stimulation.

## Results

### BMMSCs maintained their morphology after ES

**Figure 1:**
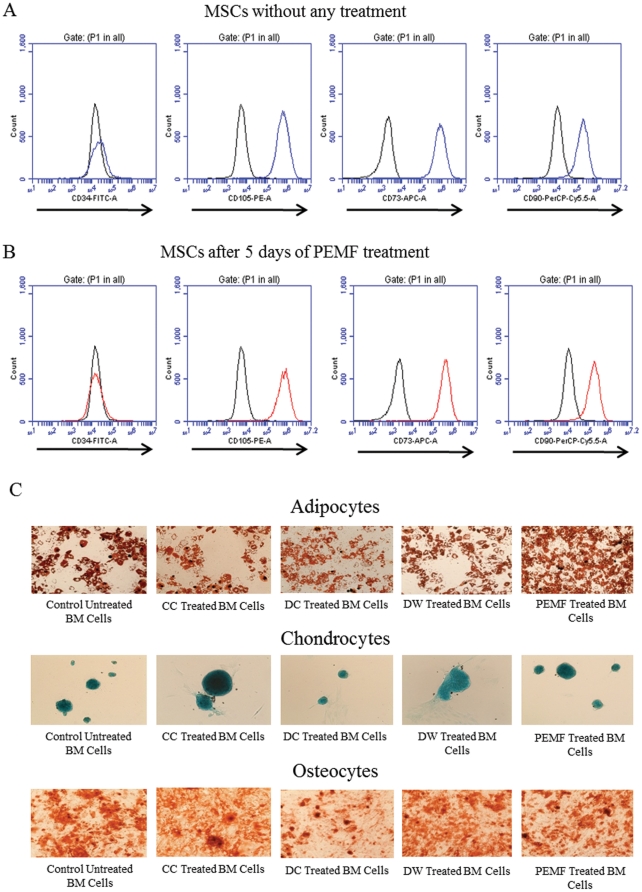
FACS analysis of Mesenchymal Stem cells (MSCs) with and without electrical stimulation. A, MSCs from bone marrow were harvested and at passage 3 were analysed for CD34, CD73, CD90 and CD105 expression. Mouse anti-human primary antibodies conjugated with fluorophores were used to label the cells. More than 90% of the MSCs were positive for CD73, CD90 and CD105 and were negative for CD34. B, PEMF treated MSCs showed the same morphology as untreated MSCs and therefore there is no effect of electrical stimulation on MSC marker expression. Similar results were obtained for DC, DW and CC treated cells (not shown here). Black line  =  Isotype control; Coloured lines  =  Labelled cells. C, MSCs tri-lineage assay confirming the differential potential of cells obtained from bone marrow. Cells differentiated into all three lineages of osteocytes, chondrocytes and adipocytes with or without electrical stimulation. Total magnification for each figure  = 100x.

In order to ascertain whether ES affects the morphology of MSCs, we looked for MSC markers before and after ES in BMMSCs. In excess of 90% of the MSCs were positive for CD73, CD90 and CD105 and were negative for CD34 before and after 5 days of ES for all waveforms, similar to the untreated control BMMSCs (Fig. 1A & B). *In vitro* differentiation assay also confirmed that the BMMSCs were able to differentiate into osteocytes, chondrocytes and adipocytes both before and after electrical stimulation (Fig. 1C).

### Significant cytotoxic effect was observed on days 1 and 2 after stimulation with DC, CC and PEMF

**Figure 2:**
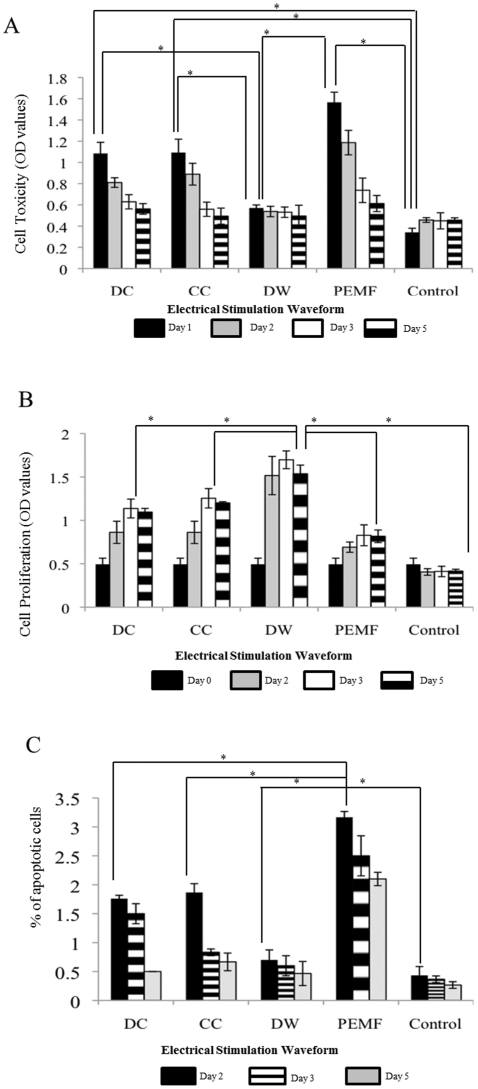
Assessment of cytotoxicity and Proliferation in BMMSCs before and after ES. A, LDH assay to assess toxicity of the BMMSCs after ES. DW has the least cytotoxic effect on cells at days 1, 2, 3 and 5 compared to the other waveforms. All waveforms showed similar cytotoxicity by the fifth day to unstimulated cells. *; p<0.001. B, Proliferation of the Bone Marrow Stem Cells (BMMSCs) was assessed by WST-1 assay. DW has the greatest proliferative effect on cells after 5 days of stimulation (DW vs. CC, p<0.01, DW vs. DC, p<0.01, DW vs. PEMF, p<0.001, DW vs. Control, p<0.001). C, Percentage of apoptotic cells shown here after days 2, 3 and 5 days of electrical stimulation. PEMF had the highest number of apoptotic cells after 2, 3 and 5 days of ES compared to the other waveforms. *; p<0.001.

The LDH enzyme activity in the cell growth medium was measured to evaluate cytotoxicity as shown in Fig. 2A. On days 1 and 2, there was a significant increase in the cytotoxicity of the cells exposed to DC, CC and PEMF compared to DW and unstimulated BMMSCs (p<0.01). By the 3^rd^ and 5^th^ day, there was a decrease in cell cytotoxicity for cells exposed to DC, PEMF and CC to a level similar to DW and unstimulated BMMSCs.

### Proliferation of BMMSCs increased significantly by the 3^rd^ day for all electrical stimulations compared to the unstimulated BMMSCs

The desired effect of ES is to increase the proliferation of progenitor cells. Therefore, we explored the proliferation of cells after 2, 3 and 5 days of ES and observed a significant increase in the proliferation of the stimulated BMMSCs after all forms of ES compared to the unstimulated BMMSCs (p<0.001) (Fig. 2B). On days 2, 3 and 5; a significant (p<0.001) increase in proliferation was observed for DW treated BMMSCs compared to other ES and unstimulated BMMSCs. Furthermore, there was a marked difference in proliferation of BMMSCs exposed to DC and CC compared to PEMF and unstimulated cells on days 3 and 5 (p<0.01).

### DW treated cells showed least apoptosis at every time point compared to other ES treatments

In order to examine whether repeated ES has any adverse effects on cell viability, we performed apoptotic assays after days 2, 3 and 5 following ES treatment. A significant number of apoptotic cells (p<0.01) were observed for BMMSCs exposed to PEMF compared to all other modes of ES at the end of days 2, 3 and 5; this steadily decreased by day 5 but did not reach the same level as BMMSCs exposed to other ES (Fig. 2C). Furthermore, CC and DC showed significantly greater number of apoptotic cells compared to DW on days 2 and 3 (p<0.05). By the fifth day, there was no significant difference in apoptosis between DW, CC and DC and unstimulated BMMSCs.

### A higher number of cells were in the S phase of the cell cycle post-ES compared to unstimulated cells

**Figure 3:**
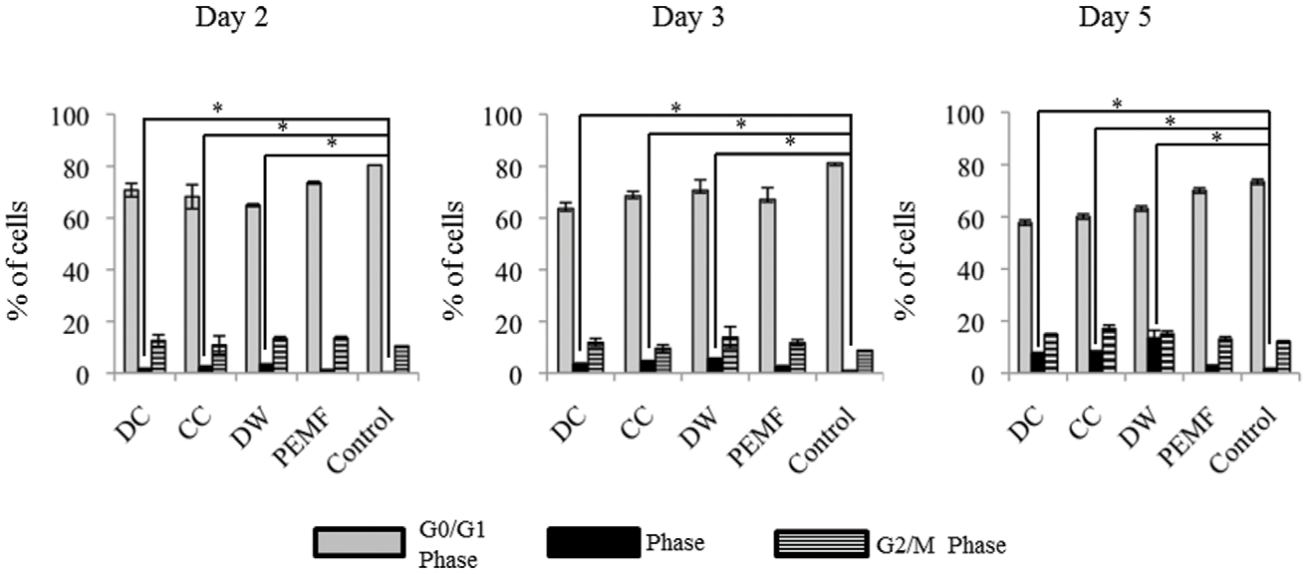
Effect of ES on cell cycle progression of BMMSCs. After 5 days of stimulation of either PEMF, CC, DW or DC there was significantly less BMMSCs in the G0/G1 phase and more in the S and M2/G2 phase compared to the unstimulated BMMSCs (p<0.001 for CC, DW and DC compared to unstimulated BMMSCs).*; p<0.001.

For investigating the effect of ES on cell cycle, we performed Propidium iodide (PI) based cell cycle analysis after 2, 3 and 5 days of ES (Fig 3). At the end of days 2 and 3, there were significantly more BMMSCs in the S phase instead of G_1_/G_O_ phase following exposure to DW, DC and CC stimulations compared to the unstimulated BMMSCs (p<0.01), although, the same effect was not seen after PEMF stimulation. By day 5, there were significant numbers of cells in the S phase and M/G_2_ phase after DW, DC and CC stimulations compared to unstimulated BMMSCs and a decrease in G_1_/G_0_ phase cells was observed.

### After 5 days of DW and CC stimulation significantly more cells invaded through the collagen membrane compared to non-stimulated control cells

**Figure 4:**
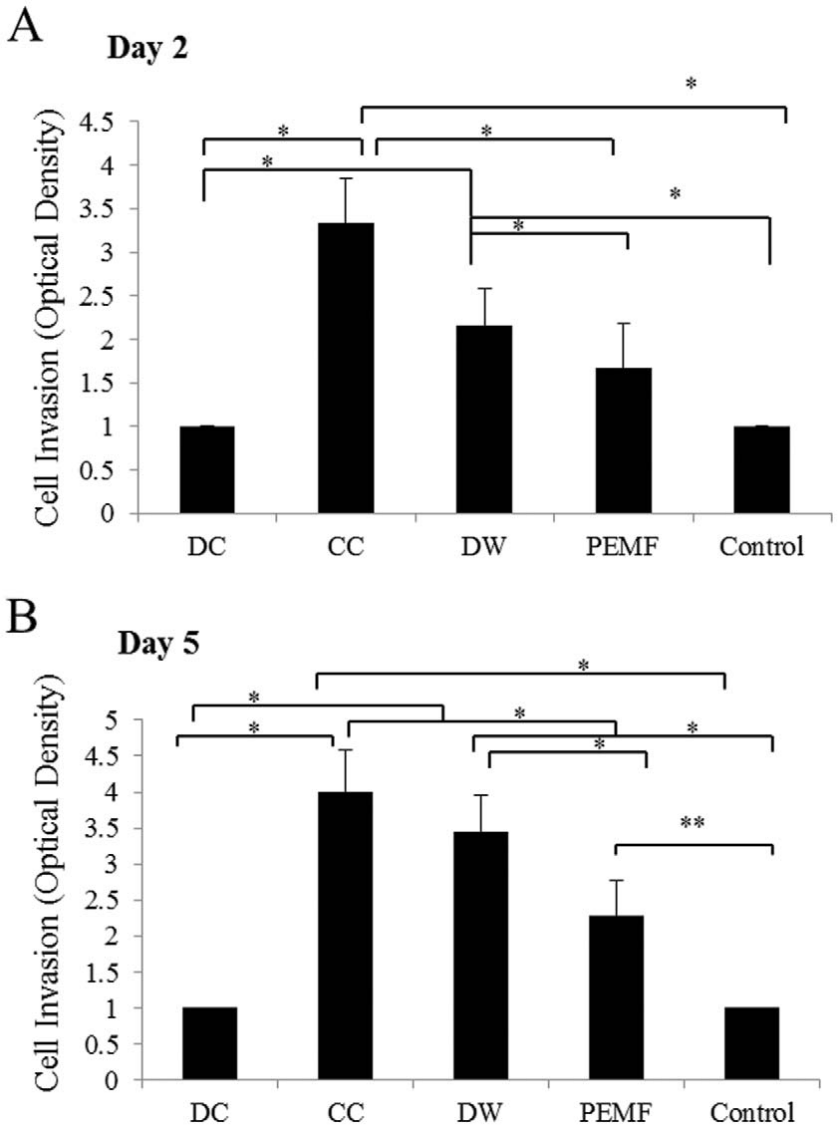
Invasion of collagen coated membrane of marrow stem cells (BMMSCs). A, After 2 days of stimulation DW treated cells showed a significantly greater number of invading cells through the collagen membrane compared to the other waveforms (p<0.001). B, After 5 days of stimulation DW and CC caused significantly (p<0.001) more BMMSCs to invade the collagen membrane.*; p<0.001.

To invade the wounded region, cells have to pass through the ECM barrier [20]. In order to verify the effect of ES on cell invasion, we investigated the mobility of cells through collagen membrane after application of ES. We found that after two days of DW and CC stimulations, significantly higher number of cells invaded the collagen membrane compared to other ES and unstimulated cells (p<0.01) (Fig. 4). There was no difference in the invasive capacity of cells treated with PEMF and DC compared to unstimulated cells on day 2. By day 5, the number of BMMSCs exposed to DW and CC invading the collagen membrane was increased compared to other modes of ES (p<0.01). However, there was no statistically significant difference for cells exposed to DC compared to unstimulated BMMSCs (p<0.78). Significantly more PEMF treated cells invaded the membrane compared to the unstimulated cells by 5th day of stimulation (p<0.05). There was also no difference in invasive capacity of cells grown in medium with or without supplementation with fetal bovine serum.

### DC stimulation resulted in the highest rate of cell migration

**Figure 5:**
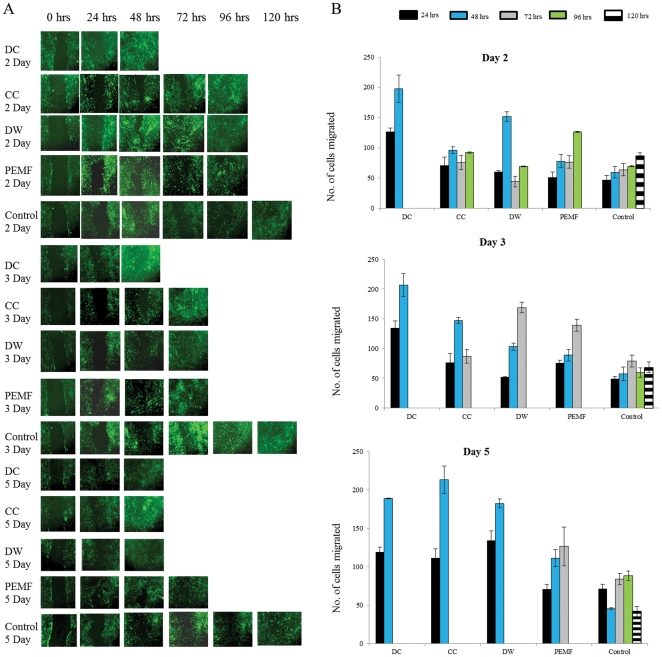
Migration of the Bone marrow stem cells (BMMSCs) into the scratch wound after 2, 3 and 5 days of electrical stimulation. A, Images of scratch wound taken after 2, 3 and 5 days of electrical stimulation. By five days of stimulation DC, CC and DW covered the scratch in 48 hours, PEMF in 72 hours and control cells in 130 hours. Total magnification  = 100x. B, Rate of movement per 24 hours after 2, 3 and 5 days of stimulation. The rate of movement was significantly greater for DC treated cells compared to the other waveforms after 2 and 3 days of stimulation (p<0.001), although by day 5 DC, DW and CC treated cells showed similar migration rates.

Electrical stimulation has previously been demonstrated to mobilize cells in the direction of cathode compared to unstimulated cells [21]. In order to identify whether there is a difference in the mobility of cells due to different ES treatments, we performed a scratch wound assay. We observed cell movement in the direction of the wound after every ES and in particular, we observed an accelerated movement for cells undergoing DC stimulation. DC treated cells took 48 hrs to fill in the scratch wound compared to CC, DW and PEMF treated cells that took between 96–120 hrs (Fig. 5). Through time lapse microscopy, we found that migration by DC treated cells was mainly due to cell movement while DW and other ES treated cells filled the gap partly through migration and partly by cell proliferation.

### DC induced migration and invasion related genes more than other ES treatments

**Figure 6:**
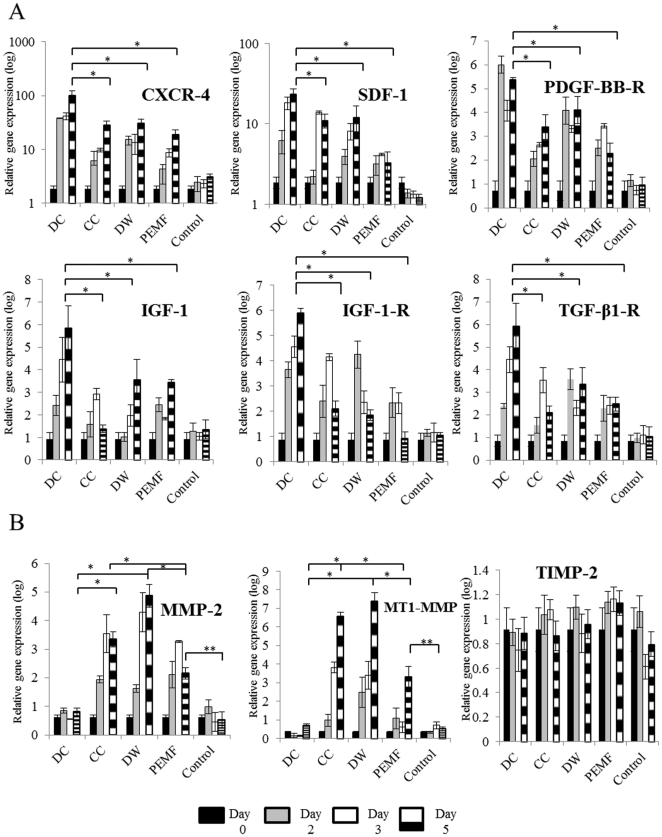
Effect of electrical stimulation (ES) on gene expression. A, Quantitative-RT-PCR analysis of migration genes after 2, 3 and 5 days of stimulation. ES increased the expression of the migratory genes with DC treated Bone marrow stem cells (BMMSCs) showing a significant increase in expression of SDF-1, CXCXR4, PDGF-BB-R, TGF-β-1-R, IGF-1 and IGF-1-R compared to other waveforms (p<0.001) on day 5. B, Quantitative-PCR analysis of invasion genes after 2, 3 and 5 days of ES. CC, DW and PEMF increased the expression of MMP-2 and MT1-MMP (p<0.001) compared to unstimulated BMMSCs and PEMF cells after 5 days of stimulation. DC treated cells showed similar expression of MMP-2 and MT1-MMP compared to unstimulated BMMSCs. TIMP-2 expression remained constant for unstimulated and treated BMMSCs. **; p<0 .05, *; p<0.001. SDF-1; Stromal Derived Growth Factor-1, PDGF -BB-R; Platelet derived growth factor beta receptor, TGF-β1-Transforming growth factor-1 receptor, IGF-1;Insulin growth factor-1, IGF-1-R;Insulin growth factor-1 receptor, MMP-2; Matrix Metalloproteinase 2, MT1-MMP;Membrane Type 1- Matrix Metalloproteinase, TIMP-2;Tissue inhibitor of matrix metalloproteinase-2.

Increased expression of stromal cell derived factor-1 (SDF-1) has shown to increase cellular migration rate [22]. We measured the expression of the receptor/ligand pair CXCR4 and SDF-1 in BMMSCs treated with ES and found that it was higher on days 2, 3 and 5 after ES compared to the unstimulated BMMSCs (Fig. 6A). BMMSCs exposed to DC stimulation showed significantly higher expression of CXCR4 and SDF-1 on days 2, 3 and 5 compared to other ES (p<0.001).

The PDGF-BB and its receptor PDGF-BB-R has been shown to stimulate migration of MSCs [23]. When we investigated the PDGF-BB-R expression, we found it was increased significantly in DC treated cells after ES on days 2, 3 and 5 compared to unstimulated BMMSCs and other ES (p<0.01) (Fig. 6A). PDGF-BB was not expressed by unstimulated or stimulated BMMSCs.

Transforming growth factor- β1-receptor (TGF-β1-R), insulin growth factor-1 (IGF-1) and its receptor IGF-1 receptor (IGF1R) have been shown to be stimulators of MSCs migration [24], [25]. We found an increased expression of TGF-β1-R for electrically stimulated cells compared to the unstimulated BMMSCs, with DC showing the greatest effect (p<0.01) (Fig. 6A). TGF-β1 was expressed before and after stimulation but showed no significant difference after ES compared to unstimulated BMMSCs. The expression of IGF-1 and IGF1R was higher on every time point after ES compared to the unstimulated BMMSCs, with DC stimulation having the greatest effect (p<0.001) (Fig. 6A).

Gene expression of MMP-2 and MT1-MMP, which are responsible for the invasive capacity of the BMMSCs, was increased to the greatest extent by cells exposed to DW and CC stimulation (Fig. 6B). There was also a significant increase in expression of these genes after 5 days of PEMF stimulation compared to the unstimulated BMMSCs (p<0.05). The expression of tissue inhibitor of matrix metalloproteinase-2 (TIMP-2) was similar on all days after all applied forms of ES and unstimulated BMMSCs (Fig. 6B). Expression of MMP-9 was barely detectable after all forms of ES and by the unstimulated BMMSCs.

### DC stimulated BMMSCs express SDF-1 and CXCR4 significantly more than other ES treatments

**Figure 7:**
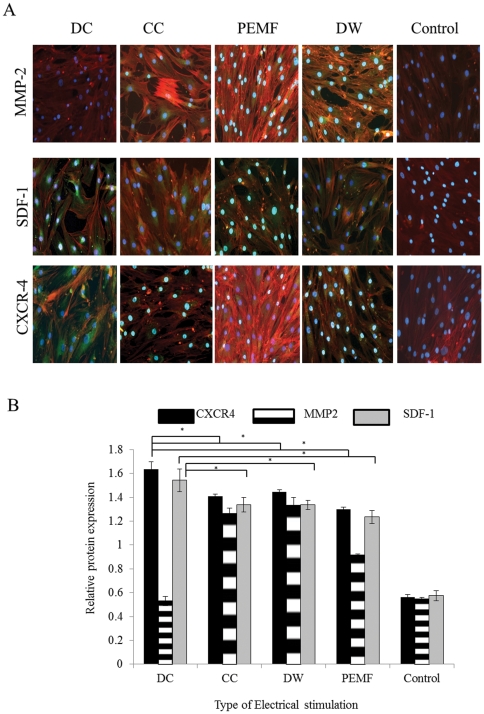
Immunocytochemical detection of cell invasion related markers. A, Immunocytochemical detection of CXCR4, SDF-1and MMP-2 after 5 days of electrical stimulation (ES). CXCR-4 and SDF-1 (Green) were expressed by the Bone marrow stem cells (BMMSCs) after all forms of ES but to the greatest extent by DC stimulation. Expression of MMP-2 (Green) was increased for BMMSCs exposed to DW and CC stimulation but not for DC or the unstimulated BMMSCs. In all samples actin filaments were stained with phalloidin (red) and the nuclei were stained with DAPI (Blue). Magnification  = 100x. B, In-cell Western blotting analysis of CXCR4, SDF-1 and MMP-2 after 5 days of ES. CXCR4 and SDF-1 protein expression were increased by all forms of ES compared to unstimulated BMMSCs with DC showing the greatest expression (p<0.001). MMP-2 expression was significantly increased by BMMSCs (p<0.001) exposed to DW and CC compared to unstimulated BMMSCs and cells stimulated with DC. SDF-1; Stromal Derived Growth Factor-1, MMP-2; Matrix Metalloproteinase 2. *; p<0.00.1The green fluorescence shows the protein names, the red fluoresence shows the Rhodamine phalloidin stain and the blue fluoresence shows the nuclear stain.

Using immunocytochemistry, we confirmed that after five days of stimulation BMMSCs showed increased expression of SDF-1 and CXCR4 with all forms of ES compared to the unstimulated BMMSCs, with DC showing the greatest expression (Fig. 7A). Using In-cell Western blotting, we quantified the protein expression of CXCR4 and SDF-1 after 28 hours of ES and found that DC treatment resulted in higher expression of both proteins compared to other ES (p<0.01) (Fig. 7B).

### Protein expression of MMP-2 was greatest after DW and CC stimulation

The protein expression of MMP-2 was greatest by day 5 after DW and CC stimulation compared to other ES, which was confirmed by immunocytochemistry. PEMF treated cells also showed greater MMP-2 expression compared to unstimulated cells. DC treated cells showed no difference in MMP-2 protein expression compared to unstimulated cells (Fig. 7A). Using in-cell Western blotting DW and CC treated BMMSCs showed the highest expression of MMP-2 after 28 hours of ES compared to DC treated cells which showed similar protein expression values compared to unstimulated BMMSCs (p<0.01) (Fig. 7B).

## Discussion

To our knowledge, this is the first study that has compared the effects of various ES waveforms on BMMSCs cellular activities *in vitro* that are relevant to bone regeneration. In this study, there was a varying effect on the BMMSCs cellular activities by the different electrical waveforms. In addition, others including our own findings (unpublished data) have shown varying effects on the proliferation, differentiation and mineralization of osteoblasts following application of different forms of electrical stimulation [26]. Therefore, the specific mode of electrical stimulation may have a significant differential effect on cellular activity.

We used BMMSCs to assess the effect of various ES as it is considered to be one of the major cell types involved in wound healing and especially because it plays an important role in bone repair [6], [27], [28], [29]. We used stromal cells from bone marrow that were found to be more than 90% positive for CD73^,^ CD90 and CD105 and negative for CD34 (Fig. 1A). Furthermore, we examined the differentiation capacity of the cells we used before or after ES and identified that all of them differentiated into characteristic MSC lineages (Fig. 1B).

All waveforms were found to increase the proliferation of the electrically stimulated BMMSCs compared to the unstimulated BMMSCs. However, BMMSCs stimulated with DW showed the greatest proliferation (Fig. 2B). We found that more cells entered the synthesis phase and fewer cells were in the G0/G1 phase after 5 days of ES. We postulate that the increase in number of cells in the S phase accounts for the increase in the proliferative activity of the MSC post electrical stimulation (Fig. 3). Several reports have shown that electrical stimulation enhances proliferation of various cells. Capacitive coupling was shown to enhance proliferation of osteoblasts by the release of IGF-II [30].

The cytotoxicity and apoptosis assays confirmed the above findings and we found proportionately less cytotoxic and apoptotic cells after DW treatment compared to other stimulations (Figs. 2A–C). There was an increase in cytotoxicity and an increase in the number of apoptotic cells on days 2 and 3 for DC, CC and PEMF treated cells compared to the unstimulated cells. DW was the only treatment that enabled the cells to proliferate from the start to the end of the stimulation without considerable cytotoxic effects. Our results also showed that stimulating MSCs for 3 hrs per day had minimal short-term cytotoxic effects on cell proliferation and even after 5 days of treatment, cells showed no significant signs of apoptosis.

MSCs have been shown to migrate towards the site of injury where they differentiate into osteocytes, chondrocytes and adipocytes depending upon their interactions in the wound [11], [12], [13], [14], [31], [32]. Electrical stimulation is known to act as a migratory stimulus for fibroblasts, macrophages and corneal epithelial cells [33]. In addition, when electric currents were applied to wounded tissue, there was an increase in the neutrophil and macrophage migration rate [34]. We found that DC treated BMMSCs migrated more than any other ES treated cells (Fig. 5). Since DC treated cells were less proliferative compared to other ES (Fig. 2A), we therefore concluded that the higher migration rate observed for DC are solely based on cell movement rather than cell proliferation.

DC treated cells also significantly over expressed several migratory genes including SDF-1/CXCR4, PDGFB-R and TGF-β1-R, IGF-1 and IGF-1R (p<0.01). It has been shown previously that FGF-2 expression was increased after PEMP treatment in diabetic mice that resulted in increased wound healing [35]. Also IGF-II expression was found to increase following low voltage electrical stimulation in bone cells which eventually resulted in cell proliferation in an in vitro model [30]. Beside these few studies, a causal link between increased gene expression and cell proliferation after ES has not been established. In previous reports, DC was shown to alter the migration of cells by affecting the actin arrangement in vascular endothelial cells [21]. Increasing evidence shows that SDF-1 is up regulated at sites of injury and is a chemo-attractant to recruit circulating or CXCR4 expressing MSCs to heart, liver, brain and skin [36], [37], [38]. Similarly, SDF-1 was shown to increase the motility of cells by rearrangement of their cytoskeletal proteins and an increased number of F-actin bundles [39].

SDF-1 has been shown to work in an autocrine manner, which may explain the higher expression of CXCR4 than SDF-1 after ES by the BMMSCs in this study [40]. Additionally, the limited expression of PDGF-BB observed in our experiments by the BMMSCs has been previously reported [21].

Osteoblasts, chondrocytes, inflammatory cells and MSCs secrete PDGF to recruit MSCs to the lesion site to promote fracture healing. Several studies have illustrated that PDGF-BB is a potent stimuli for MSCs [23], [41]. PDGF-BB-R and TGF-β1-R were up regulated by ES but their ligands were not altered by ES. This finding is still important as PDGF-BB and TGF-β1 are secreted by several inflammatory cells during the early stages of fracture healing to recruit MSCs to the fracture site [5] and therefore up regulation of the expression of their receptors may result in an increased sensitivity of MSCs to TGF-β1/PDGF-BB signaling.

IGF-1 was up regulated by day 5 in DC, DW and PEMF treated cells compared to the unstimulated cells. Although IGF-1 has been demonstrated to recruit BMMSCs to the fracture site during the early stages of healing *in vivo*, it has been recently shown that IGF-1 increases the migration of BMMSCs *in vitro* and can mediate its effect through the CXCR4-SDF-1 axis [24], [42]. Therefore, if ES can increase the expression of these migratory genes in MSCs, then it could also recruit these cells *in vivo* for bone regeneration. The understanding of the migratory capacity of MSCs post ES is important as modulation of their activity by ES could increase the efficiency of MSC homing into damaged tissues and organs.

Using an invasion assay, which employs a collagen membrane that mimics the natural ECM [43], the invasive capacity of cells treated with CC and DW was found to increase, which correlates with the highest expression of MMP-2 and MT1-MMP after CC and DW as these genes have been shown to be associated with invasion of BMMSCs. It has been shown that when MT1-MMP was knocked down, the invasive capacity of human mesenchymal stem cells (hMSCs) was found to be blocked when invading through a modified matrigel membrane made of rich ECM proteins [42]. Furthermore, it has been reported that the knockdown of MMP-2 and MT1-MMP impaired hMSCs invasive capacity through a reconstituted human basement membrane [43]. The MMP/Tissue Inhibitor of Matrix Metalloproteinase (TIMP) balance is important in cellular invasion [15] as TIMP-2 regulates the expression of MMP-2. High expression of TIMP-2 decreases the activation of pro-MMP-2 by MT1-MMP [43]. TIMP-2 remained constant after ES compared to the unstimulated cells, which may account for the increase in expression of MT1-MMP. The limited expression of MMP-9 by BMMSCs in this study has also been documented in previous studies [43].

Invasion of tumor cells has been associated with increased expression of MMP-2 [40], [44]. However, the morphology of the BMMSCs in this study did not change after ES and the increase in proliferation was similar after days 2, 3 and 5. DC showed the highest migratory capacity using the scratch wound assay, as it could not invade through the collagen membrane. This illustrates that DC was not efficient in migrating through a barrier, which, may be associated with the lack of expression of the MMP2, required to break down the barrier (Fig. 6).

Through this study, we have found that cells treated with DW and CC stimulations showed higher proliferation, less apoptosis and higher cell invasion through collagen barrier compared to DC, PEMF and untreated cells. However, DW was found to be the most optimal ES in this *in vitro* study as it had the greatest proliferative, least cytotoxic and least apoptotic effects. In addition DW enhanced the migration and invasion of the BMMSCS.

In view of the above findings, further investigation into DW and CC would be of clinical relevance in order to better characterize their application in tissue regeneration. Moreover, further *in vitro* and animal studies are needed to understand the mechanistic pathways involved in BMMSC migration and invasion post ES. Better understanding of MSC homing capacities using ES would be ideally utilized to engineer BMMSCs capable of enhancing tissue regeneration. In conclusion, we provide evidence that different ES waveforms affect BMMSCs cellular activities including cell proliferation, migration, apoptosis and barrier invasion.

## Materials and Methods

### Isolation of BMMSCs and Cell Culture

Bone marrow samples were obtained, after gaining informed consent and written approval from Thameside and Glossap Ethical Committee (rec 09/h1013/38) U.K., from femur of six male patients (mean age 60 years range 54–79 years), who were undergoing hip replacement surgery. The University of Manchester Ethics committee duly endorsed the above written ethical approval. Written informed consent was taken from each patient undergoing surgery. In all cases, bone marrow mononucleated cells (MNCs) were isolated by Ficol-paque (Stem Cell Technologies, France) density gradient centrifugation within 8 hours of extraction. The MNC fraction was further washed with PBS and a cell count was performed. The BMMSCs were plated in 100 mm^2^ petri plates in Minimum Essential Medium Eagle (MEM) (Sigma-Aldrich Aldrich, UK) supplemented with 20% fetal bovine serum (PAA, Austria), Penicillin (100 units/ml), streptomycin (100 units/ml) and L-Glutamine (2 mM, PAA Austria). They were incubated and grown to confluence in 0.2 µm vented T75 flasks (Corning Incorporated, USA) at 37°C in humidified air with 5% CO_2_ before being passaged once 80% confluent (4 days) using TrypLE Express (Invitrogen, UK). Passage numbers 3–10 were used for experiments.

### Slides for cell growth

Large microscopic cover slips (22×50 mm^2^) were divided into four using a diamond tip pen. Resulting smaller cover slips had dimensions of approximately 11×25 mm^2^ and are referred to as slides. These slides were sterilized by autoclaving before seeding cells. After seeding 1.5×10^4^ cells per slide it generally took 18–24 hours for the cells to stick and spread on the surface.

### Equipment and settings used for Electrical stimulation

**Figure 8:**
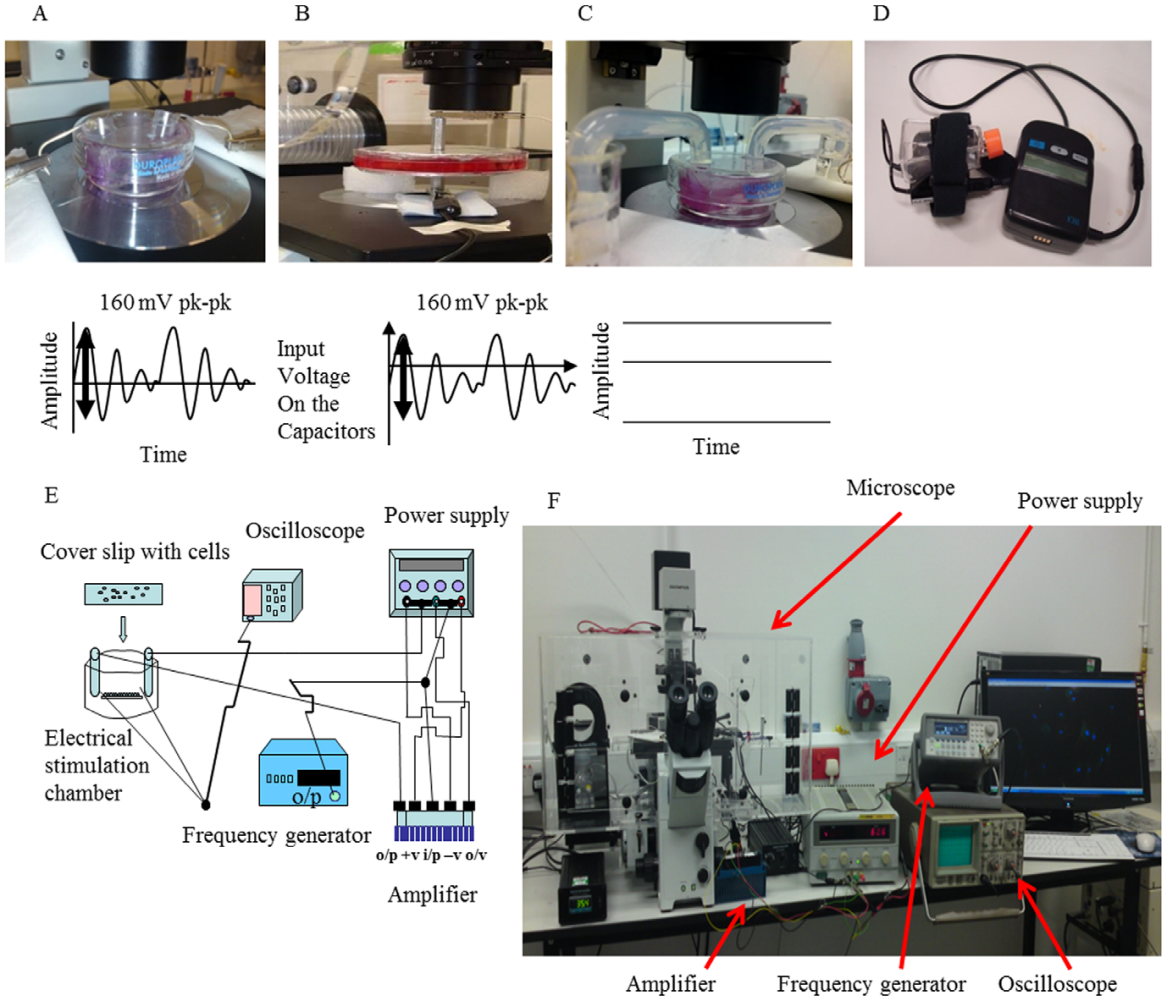
Setup for electrical stimulation used in this study. A, Electrical stimulation (ES) chamber used for Degenerate Wave and the Degenerate waveform. B, ES apparatus for Capacitive Coupling (CC) and the CC waveform. C, ES apparatus for Direct Current (Direct Current) and DC Waveform. D, Pulsed Electromagnetic field stimulation (PEMF) device. E, Schematic diagram of the ES apparatus used for DC, CC and DW. F, Photograph of the microscope incubator where the ES stimulation takes place for CC, DW and CC and where control cells are placed.

The overall setup for ES is shown in Fig. 8. To deliver DW current to a monolayer of BMMSCs a precise ES apparatus [Fig. 8A] was designed as used previously [19]. The DW current was programmed for 15 Hz (62.5 ms pulse width) and 160 mV peak-to-peak on the Agilent function generator at a constant current of 0.3 mA, which delivered an electric field of 10 mV/mm between the electrodes. For CC a similar set up to Hartig et al. was used [45], see supplementary data S1. Voltage peaks of 0.16 µV were observed across the cell monolayer when 160 mV peak-to-peak was applied to the capacitors [Fig. 8B], which gave a desired resultant electric field of 10 mV/mm across the cell membrane at a current of 0.6 mA, which was observed with the oscilloscope. To stimulate the cells with DC a similar set up [Fig. 8C] was used as described previously [46].

To stimulate the cells with PEMF the same system was used as Sun et al [47]. The PEMF device (Biomet EBI Bone healing system, Strawberry Medical UK) was placed in the incubator at 37°C and 5% CO_2_ [Fig. 8D]_._ The applied field consisted of 4.5 ms bursts of 20 pulses repeating at 15 Hz. During each pulse, the magnetic field increased from 0 to 1.8 mT in 200 ms and then decayed back to 0 in 25 ms. BMMSCs were cultured in T25 flasks using an initial seeding density of 8×10^4^ BMMSCs per flask to be connected to the PEMF device.

### Protocol for electrical stimulation

For DC, CC and DW the required number of cell grown slides were placed in the electrical chamber. After applying either DW, DC, CC or PEMF the BMMSCs were stimulated for 3 hours a day for five days with rest periods of 21 hours where the BMMSCs were kept in the 5% CO_2_ incubator at 37°C overnight in small petri-dishes. At certain days, slides were extracted to assess the effect of the different waveforms on cellular activities using various techniques. The zero time point was before any ES was applied. In the same microscope incubator box (Fig. 8E–F) along with the cells undergoing ES the same number of slides were placed in the incubator box under the same conditions, but without ES as control.

### Fluorescent Activated Cell Sorting (FACS) analysis

For FACS analysis bone marrow cells were harvested from passage 3 (P3) or from electrically stimulated cells at the end of day 5 using TrypLE Express. The procedure is the same as described earlier in Iqbal et al. [48]. Briefly 2×10^5^ cells were rinsed twice with 5%FBS/HBSS and were incubated with primary antibodies (1∶50 dilution), CD34-FITC (BD Biosciences, USA), CD73-APC, CD90-PerCP-Cy5.5 and CD105-APC (eBioscience, UK), all mouse anti human, for 30 minutes on ice. Propidium iodide (PI; Sigma-Aldrich, UK) was added to determine cell viability. To assess non-specific binding, isotype-matched primary antibodies conjugated to respective fluorophores were used as controls. FACS was performed on Accuri C6 (Accuri Cytometers, USA) equipped with 2 lasers. The data was analyzed and presented using C6 Plus Analysis software package (Accuri Cytometers, USA).

### Cell viability and toxicity

The cell toxicity was determined by measuring the secretion of Lactate dehydrogenase (LDH) by the necrotic cells into the growth medium and was performed as per manufacturer's instruction (Cytotoxicity Detection Kit, Roche Mannheim, Germany).

### Cell proliferation

The effect of ES on cellular proliferation was determined by WST-1 assay (Sigma-Aldrich CO, UK) as per manufacturer's instructions. The assay is based on the reduction of WST-1 by metabolically active cells to produces a soluble formazan salt, which was read between 420–480 nm wavelength on a microplate reader (BMG Labtech, Germany).

### Cell apoptosis

After ES apoptosis was quantified using an Apoptotic Blebs Assay kit (Accuri Cytometers, UK) as per manufacturer's instructions. The kit “employs a recombinant protein containing single-chain variable fragments (scFv) of D56R fused to the protein A domain B fraction as an antibody for apoptotic blebs.”

### Cell cycle analysis

Following electrical stimulation BMMSCs were harvested after trypsinisation using TrypLE Express and were fixed in 2.5 mL of absolute ethanol on ice for 30 min. Cells were washed once in PBS followed by incubation at 37°C for 40 minutes with PI staining solution (50 µg/mL) supplemented with RNase A (0.1 mg/ml; Qiagen, UK) (and triton X-100 (0.05%; Sigma-Aldrich, UK). The cells were washed once and reconstituted in 500 µl PBS and analyzed on Accuri C6 cytometer.

### Cell invasion

In order to assess the invasive properties due to ES, cells were harvested at the end of 5^th^ day of ES treatment and transferred to collagen I coated inserts in a 24-well plate, part of the Collagen I invasion assay kit (Millipore, UK). The method is based on Boyden chamber principle whereby cells that passed through the collagen I matrix are stuck to the underside of the insert, which was washed and detached cells were labeled with fluorescent dye and quantitated on a microplate reader (BMG Labtech, Germany).

### Cell scratch wound assay

For the scratch wound assay cells were labeled with PKH2 (Green membrane fluorescent dye; Sigma-Aldrich, UK) and electrically stimulated as described above. After 2, 3 and 5 days of ES 4×10^4^ BMMSCs were grown to confluence, which took an average of 18 hours in a 24-well plate in triplicates. Vertical scratches were then made using a 200 µl plastic filter tip to create a ‘wound’ of approximately 200 µm in diameter. To eliminate dislodged cells, culture medium was removed and wells were washed with PBS. The number of new cells that had moved into the scratch was counted every 24 hours and digital images were taken until the BMMSCs filled the scratch wound on an inverted Microscope (IX71, Olympus UK Ltd.).

### Quantitative Real Time-PCR

Following electrical stimulation cells from the slides were harvested using TRIzol (Invitrogen, UK) and then disrupted in QIAshredder columns (Qiagen, UK). RNA was extracted using RNeasy kit (Qiagen, UK) according to manufacturer instructions. Transcriptor first strand cDNA synthesis kit (Roche applied science, UK) was used to synthesize cDNA. The cDNA (1∶20 dilution) was used to perform probe based qRT-PCR on LightCycler 480 machine (Roche Diagnostics, Germany). The primers used for the amplification of specific genes are shown in table 1.

**Table 1:** Gene specific primers sequence data.

Target Gene	Primer Sequence (bp)
CXCR4	Forward: aaggtgtgattgagaacgReverse: acaccttgcttgatgatttc
SDF-1	Forward: cgattcttcgaaagccatgtReverse: cctccagaagaggcagac
TGF-β1-R	Forward: ccaaaccacagagtgggaacReverse: caggggccatgtacctttt
PDGF-BB-R	Forward: cccttatcatcctcatcatgcReverse: ccttcatcggatctcgtaa
IGF-1	Forward: gcagtcttccaaccaatgReverse: cccattgcttctgaagtcacaaa
IGF-1-R	Forward: aaaaacctccctcatcctReverse: tggttgtcgaggacgtagaa
MMP-2	Forward: ctgagggcgctctgtctcReverse: ctgagaagtcacttgcctaacatc
MT1-MMP	Forward: tacttcccaggccccaacReverse: gccaccaggaagatgtcatt
TIMP-2	Forward: gaagagcctgaaccacaggtReverse: cggggagggtgtagcac
RPL32	Forward: gaagttcctggtccacaacgReverse: gagcgatctcggcacagta9)

Abbreviations: SDF-1: Stromal Derived Growth Factor-1, HGF: Hepatocyte growth factor, TGF-β1: Transforming growth factor-1 receptor, PDGF-BB-R: Platelet derived growth factor beta receptor, IGF-1: Insulin growth factor-1, IGF-1-R: Insulin growth factor-1 receptor, MMP-2: Matrix Metalloproteinase 2. MT1-MMP; Membrane Type 1- Matrix Metalloproteinase. TIMP-2, ; Tissue inhibitor of matrix metalloproteinase -2.

Delta Ct was determined by subtracting the averaged Ct of the reference gene RPL32 from the Ct of the target genes, with relative gene expression levels being calculated by using 2^-ΔΔCt^ method [49].

### Immunocytochemistry

After electrical stimulation cells were fixed in 10% neutral buffered formalin, washed in PBS and then permeabilised with 0.1% Triton X-100 in PBS. After further washing cells were incubated with blocking solution (1% BSA in 0.1% PBST). Cells after washing were incubated overnight at 4°C with either Rabbit-polyclonal anti-human MMP-2, SDF-1 or CXCR4 antibodies (abcam, UK) at a dilution of 1∶200 in PBS. After washing, cells were incubated with the secondary antibody Anti-rabbit AF-488 (1∶200; Jackson Laboratories, USA) and with Rhodamine phalloidin stain (1∶1000) (Sigma-Aldrich, UK) for 1 hour at room temperature. DAPI (Invitrogen, UK) was added and slides were mounted with Prolong gold (Invitrogen, UK). Images were taken on an upright fluorescence microscope (BX51, Olympus UK Ltd.).

### Protein quantification using in-cell Western blotting

In-cell Western blotting was carried out for β- Actin control, MMP-2, CXCR4 and SDF-1 as described in Syed et al. [50]. Briefly after permeabilsing and blocking, the cells were incubated with primary rabbit polyclonal CXCR-4, MMP-2, SDF-1 antibodies and mouse polycolonal B actin antibody (all at 1∶200) for 1 hour at room temperature. Cells were then incubated with donkey anti mouse-AF-680 secondary antibody ( 1∶800) or donkey anti rabbit IRDye-800CW (1∶800; both Abs from LI-COR, UK) in PBS/0.1% Triton X-100 for 1 hour at room temperature in the dark. The samples were imaged and quantified on Odyssey infrared scanner (LI-COR, UK) with laser sensitivity of 7.5 (arbitary units).

### Statistical Analysis

All experiments were repeated three times in triplicates except in cell Western blotting where we have performed 2 independent experiments. One Way ANOVA was performed followed by Tukey post-hoc test using Stats Direct software and p<0.05 and p<0.001 were regarded as significant and highly significant respectively.

## Supporting Information

Data S1 CC apparatus.
(DOC)
