# Supplementary material for: Degenerate Wave and Capacitive Coupling Increase Human MSC Invasion and Proliferation While Reducing Cytotoxicity in an In Vitro Wound Healing Model
Source: PLoS One. 2011 Aug 16;6(8):e23404. doi: 10.1371/journal.pone.0023404 (PMC3156742; doi:10.1371/journal.pone.0023404)
Supplement: Data S1 — CC apparatus. (DOC) [file pone.0023404.s001.doc]

Supplementary data S1

**CC apparatus**

Plastic petri dishes (35 mm x 10 mm) were used for subjecting cell-grown slides to CC. The required number of cell grown slides were placed in the petri dish before any attachment of the chamber to electrical equipment. The capacitors were connected according to figure 1C and the remaining electrical connections were set afterwards. Capacitors consisted of a pair of high-grade steel electrodes, each with a diameter of 8 cm, mounted in a plastic insulating material. The petri dish was placed between these electrodes; the upper electrode kept approximately 2 mm above the medium leaving a small air gap and the lower electrode under the petri dish. The whole system was placed in a 5% CO_2_ controlled and 37°C temperature-regulated incubator box as explained for DW. To deliver CC, degenerate waves were generated from the frequency generator which had a pulse width of 62.5 ms and a frequency of 15 Hz. This signal was amplified and fed to the capacitors. The voltage was adjusted on the frequency generator in such a way that the capacitors received 160 mV. Voltage peaks of 0.16 µV were observed across the cell monolayer when 160 mV peak-to-peak was applied to the capacitors, which gave a desired resultant electric field of 10 mV/mm across the cell membrane at a current of 0.6 mA, which was observed with the oscilloscope and Femlab electric field simulation. A considerable voltage loss happens between the cell membrane due to the high electrical resistivity of the plasma membrane (~8 nm thickness) when compared to the cytoplasm, which the waveforms will twice counteract between the electrodes when the cell is perpendicularly oriented towards the external electric field. So, the electric field experienced by a single cell is determined as (0.16 µV / 2 x 8 x 10^-9^ ), which resulted in 10 mV/mm across the cytoplasm. The electrical parameters of the polystyrene bottom of the petri dish, cell monolayer adherent to the petri dish surface, the cell growth medium and the air space separating the upper electrode from the growth medium are taken into consideration while Femlab electric field simulation.

**Vertical section view of the capacitive coupling ES chamber**

The cells were cultured in glass cover slides and the adherent cell monolayers were placed between the pairs of electrodes constituting the capacitors. Nearly 12 slides were placed in the Petri dish and the input signal generated by the function generator was always monitored in the oscilloscope. The potential difference between the upper level and the bottom of the medium in the Petri dish was also measured with the oscilloscope and Femlab simulation. Therefore, the current passed through the cells as evident from the morphology of cells, before and after electrical stimulation when compared to the control cells and the oscilloscope readings.
